# Supplementary material for: MetaRibo-Seq measures translation in microbiomes
Source: Nat Commun. 2020 Jun 29;11:3268. doi: 10.1038/s41467-020-17081-z (PMC7324362; doi:10.1038/s41467-020-17081-z)
Supplement: Supplementary file 10 — Supplementary Data 7 [file 41467_2020_17081_MOESM10_ESM.zip › File2/Confidence_High_Taxonomy/58082_out.krona.html]

Javascript must be enabled to view this page.

members
magnitude
magnitudeUnassigned
count
unassigned
taxon
rank

58082\_out

15

15
superkingdom
2

15
phylum
976

class
200643
15

15
order
171549

815
family
15

816
genus
15

329854
species
13

SRS014948\_contig\_number\_21767SRS015960\_contig\_number\_17601SRS018623\_contig\_number\_13530SRS045004\_contig\_number\_11697SRS045645\_contig\_number\_22153SRS050422\_contig\_number\_21679SRS056259\_contig\_number\_contig-100\_552.151576SRS077194\_contig\_number\_23337SRS078242\_contig\_number\_6866SRS101376\_contig\_number\_17642SRS144362\_contig\_number\_22560SRS148319\_contig\_number\_13337SRS893295\_contig\_number\_contig-100\_7875.7876


SRS012273\_contig\_number\_33163SRS022071\_contig\_number\_9754
2
2292002
species
